# The effectiveness of a 3-week spa therapy on the 6-month mobility and functional ability of patients with knee osteoarthritis: the ANGELLO randomized controlled trial

**DOI:** 10.1007/s00484-025-02927-2

**Published:** 2025-05-13

**Authors:** Romain Forestier, Françoise Debiais, Natacha Michel, Romain Schueller, Christine Juhel

**Affiliations:** 1 Centre de recherche rhumatologique et thermal, Villa Forestier, 3 avenue d’Albion, Aix-les-Bains, France; 2 Rhumatology Department, Poitiers University Hospital, Poitiers, France; 3 Regional Staff for Ageing and Maintenance of Autonomy, Tours University Hospital, Tours, France; 4 CEN, 18 rue Pauline Kergomard, Dijon, 21000 France

**Keywords:** Knee osteoarthritis, Spa therapy, Balneotherapy, Function, Stiffness, Pain

## Abstract

Spa therapy is a potential treatment for knee osteoarthritis (KOA). The primary objective of this randomized controlled trial (RCT) was to investigate the effectiveness of a 3-week spa therapy intervention on the 6-month change in function (mobility and functional ability) of KOA patients. Secondary objectives examined the effectiveness of the intervention on KOA-related pain, stiffness, overall symptoms severity, and patient’s quality of life. Community-dwelling adults, aged 50–80 years-old with KOA were randomized to spa therapy intervention or control (usual care) group. The primary outcome measure was assessed using the Western Ontario and McMaster Universities osteoarthritis index (WOMAC) function subscale. Other outcome measures were the WOMAC pain subscale and a pain visual analog scale, the WOMAC stiffness subscale, the WOMAC total scores, the EQ-5D-3 L for quality-of-life, and patient reported outcomes. Among the 173 individuals randomized, 145 (*n* = 74 in spa therapy, mean age 67.5 ± 6.4, 67.6% women; *n* = 71 in controls, mean age 68.3 ± 6.7, 64.8% women) were analyzed. A significant between-group mean difference of 11.7 points in the WOMAC function subscale, favoring the spa therapy group, was found at six months follow-up (primary outcome measure). The intervention was effective in significantly improving most secondary outcomes, including the WOMAC stiffness, pain (and pain visual analog scale) and total scores, as well as the minimum clinically meaningful changes in KOA symptoms, and patient’s quality of life. Improvements were achieved during the intervention period and remained significant over the 6-month follow-up. This work demonstrated the effectiveness of spa therapy in KOA treatment.

**Supplementary Information:**

The online version contains supplementary material available at 10.1007/s00484-025-02927-2.

## Introduction

Osteoarthritis is one of the most burdensome chronic conditions, being the 14th cause of years-lived with disability in the world (Ferrari et al. 2024). The knee is the most prevalent site, with an age-standardized prevalence of 4307.4 cases per 100 000 in the year 2020 (GBD 2021 Osteoarthritis Collaborators 2023). Knee osteoarthritis (KOA) prevalence increases with age, reaching a peak at around 80–84 years; projections estimate that 642 million people will have KOA in 2050, which represents an increase of 74% between 2020 and 2050 (GBD 2021 Osteoarthritis Collaborators 2023). Beyond its well-known burden caused by joint pain, stiffness and, as a consequence, reduced mobility and quality of life (Castro-Dominguez et al. 2024; Courties et al. 2024; Langworthy et al. 2024), osteoarthritis, and in particular KOA, is an important economic burden, representing 1-2.5% of Gross National Product in several countries, including Canada, Australia, US, and UK. Therefore, finding out effective strategies to control pain and improve mobility and functional ability are of major importance from both individual and societal perspectives.

Spa therapy has been argued as a potential treatment for osteoarthritis (Mennuni et al. 2021; Morer et al. 2017), including KOA (Forestier et al. 2016; Mennuni et al. 2021). One of the largest randomized controlled trials (RCT) ever performed on spa therapy for KOA patients found that three weeks of spa treatment (e.g., massages, showers, mud and pool sessions) improved pain and function (mobility and functional ability) compared to controls. However, two recent reviews (Castro-Dominguez et al. 2024; Langworthy et al. 2024) do not mention spa therapy as a treatment for KOA, indicating that most KOA management guidelines recommend loss of weight, physical exercise, and pharmacological interventions (e.g., anti-inflammatory and analgesics drugs). Even though some national learning societies recommend spa therapy for treating KOA symptoms, such as French Societies of Rheumatology and Physical Medicine and Rehabilitation (Pers et al. 2024), they also recognize a low level of evidence. Similar conclusions were found by systematic reviews (Forestier et al. 2016; Fraioli et al. 2018), which suggest that spa therapy may benefit KOA treatment, in particular by reducing pain and function symptomatology, but also by improving patient’s quality of life (Antonelli et al. 2018); however, the evidence is still scarce and not robust enough to draw solid conclusions. Therefore, further RCT are needed in order to strengthen the evidence about the effectiveness of spa therapy for improving symptoms of KOA.

The primary objective of the present RCT was to investigate the effectiveness of 3-week spa therapy on the function subscale of the Western Ontario and McMaster Universities osteoarthritis index (WOMAC) (Bellamy et al. 1988) over six months in people 50-to-80 years with KOA. Secondary objectives were to examine the effects of the intervention on KOA pain intensity, stiffness and symptoms severity, clinically relevant improvements in KOA symptoms, then patients’ quality of life, as well as patients’ perception of the evolution of their KOA symptoms, evolution of concomitant treatments of KOA, utilization of KOA-related care services and presence of adverse events.

## Methods

### Study design & randomization procedures

This is a parallel-group RCT developed in the thermal center of Saint Jean d’Angély, Nouvelle-Aquitaine Region, Western, France. The RCT was approved by the Ethics Committee of Ile-de-France VI; it started in August 2020 and finished in July 2021. All the participants signed an informed consent form before enrollment in the study. The trial was registered in a publicly accessible database (https://clinicaltrials.gov/), under the name ANGELLO and the registration number NCT05819437.

Participants were randomized in a 1:1 allocation ratio between intervention and control groups. The randomization was performed in blocks of four by a statistician not involved in the statistical analysis of the trial, using a statistical software (randomly permuted blocks, SAS version 9.4), and guaranteeing allocation concealment. The study physician, and principal investigator, was not involved in the randomization process and was kept blinded to group allocation throughout the investigation; participants were asked not to reveal their group assignment.

### Study population

Inclusion criteria were: men and women; aged 50 to 80 years-old; KOA at stages 2-to-4 according to the Kellgren-Lawrence classification (Kellgren and Lawrence 1957) from images performed within the past three years; a WOMAC function subscale score ≥ 21; availability to participate in spa therapy for three weeks with a 6-month follow-up; having signed the informed consent and being affiliated to a social security regimen.

Exclusion criteria were: contra-indication to receiving spa therapy; chronic infectious disease, active cancer, heart failure, liver or kidney uncontrolled conditions; leg ulcer; behavioral symptoms; immunologic deficit; phlebitis; erysipelas or history of erysipelas; active thrombosis; chronic pain unrelated to the KOA; chronic inflammatory rheumatic disease (rheumatoid arthritis, ankylosing spondylitis, lupus, psoriatic arthritis) or fibromyalgia; scheduled surgery related to the KOA in the next seven months; having received spa therapy in the past six months; having received intra-articular injection of corticosteroid in the past 90 days or hyaluronic acid infiltration in the past six months for KOA treatment; pregnant or breastfeeding woman; woman at childbearing capacity not on effective contraception (oral, vaginal, transdermal, injectable or subcutaneous estrogen/progestogen); living more than 30 km away from the thermal center where the intervention was performed; being protected by law or deprived of liberty by administrative or judicial decision; being involved in another clinical trial.

### Intervention

The 3-week spa therapy was provided in addition to the usual care. The intervention proposed in this study follows the French agreement for thermal treatments, which is composed of three weeks of spa therapy and three consultations with a physician (at the first day of treatment, day 10, and day 20) of the thermal center providing the intervention. This physician prescribes the spa therapy composed of four daily treatments provided from Monday to Saturday (18 days of effective treatments): (1) a bath with air jets and diffusion for 10 min with water between 34 °C and 38 °C; (2) a bath with immersion shower for 10 min with water between 34 °C and 38 °C; (3) a mud poultice with a target temperature at 45 °C for 10 min on both knees and on other painful joints if patients show signs of the spread of osteoarthritis; (4) an underwater massage performed by certified physiotherapists under a mineral water affusion ramp at 38 °C for 10 min. The natural mineral water for this study is extracted from a drilling located at 975 m depth, emerging at 48.1 °C and its characteristics are: pH at 6.97, conductivity at 25 °C of 4222 µS/cm, dry residue at 260 °C of 3542 mg/L, bicarbonates 201 mg/L, chlorides 203 mg/L, sulfates 2167 mg/L, calcium 517 mg/L, magnesium 124 mg/L, sodium 377 mg/L, and potassium 61 mg/L.

### Control group

The control group is a usual care group formed by patients in the waiting list. KOA usual care is defined according to the family doctor prescriptions and may include medications, physiotherapy, and orthopedic devices. The only treatment precluded to the control group was spa therapy during the 6 months of the study. Both control and intervention groups received a booklet with general information and advice about how to improve the symptoms and progression of KOA.

### Outcome measures

All participants were evaluated at inclusion, day 20 (last day of spa intervention), three months, and six months. Participants in the intervention group, in accordance with French agreement for thermal treatment, have an additional medical consultation with the physician of the thermal center at day 10.

#### Primary outcome measure

Changes on functional ability as measured by the 17-item function subscale of the self-reported WOMAC questionnaire (Bellamy et al. 1988) between the visit at the first day of the study and the 6-month visit. Scores in this subscale vary from 0 to 68, with higher scores indicating worst function. To facilitate comparisons with previous research, a normalized score varying from 0 to 100 (higher is worse) was also used.

#### Secondary outcome measures

Changes in the outcomes between the visit on the first day of the study and the 20-day (last day of intervention), 3-month and 6-month (except for the primary outcome measure) visits. The following outcomes were assessed:

- KOA pain intensity. This was measured using two tools: the 5-item pain subscale of the self-reported WOMAC questionnaire (scores vary from 0 to 20, higher is worse) and an analog visual scale from zero (no pain) to 100 (the worst pain possible). Similar to the primary outcome measure, a normalized score varying from 0 to 100 (higher is worse) was also used.
- KOA stiffness. This was measured by the 2-item stiffness subscale of the self-reported WOMAC, with scores varying from 0 to 8, higher is worse. A normalized score varying from 0 to 100 (higher is worse) was also used.
- KOA symptoms severity. This was measured by the 24-item self-reported WOMAC index, with scores varying from 0 to 96, higher scores indicating more severe KOA symptomatology. A normalized score varying from 0 to 100 (higher is worse) was also used.
- Clinically meaningful changes in KOA symptoms. This was assessed by using either a pain reduction ≥ 19.9 mm in the 0-100 visual analog scale or a decrease of 9.1 or more in the normalized score (from 0 to 100, higher is worse) of the WOMAC function subscale (Tubach et al. 2005).
- Quality of Life. This was measured with the self-reported questionnaire EUROQOL EQ-5D-3L (Rabin and de Charro 2001) using the values for the French population (Chevalier and de Pouvourville 2013)– higher values are better.
- Patients’ perception of KOA symptoms’ evolution. This was assessed by the question: What is your opinion about the evolution of your knee osteoarthritis after 20 days/3 months/6 months: highly improved; moderately improved; mildly improved; did not change; mildly worsened; moderately worsened; highly worsened.
- Concomitant treatments for KOA. Information on all pharmacological and non-pharmacological rheumatological treatments (except those related to rheumatic pain control) were collected during the whole duration of the study. The number of individuals with at least one treatment was collected in each time-point of data collection.
- Medications for KOA pain control. All drugs for KOA pain control were collected during the whole study follow-up length. The number of individuals with at least one pain control medication was collected in each time-point of data collection.
- Consultations with a physician. The number of people who have at least one KOA-related consultation with a physician (general practitioner or specialist) was collected in each time-point of data collection.
- Adverse events. Serious and non-serious adverse events were collected over the 6-month follow-up.

### Sample size calculation

Sample size calculation was based on a previous RCT (Forestier et al. 2010) showing a difference between the control group and the spa therapy group of 5.5 points favoring the spa intervention in the function subscale of the WOMAC questionnaire at 6-month follow-up. In order to show a significant difference between control and spa therapy groups of 5 points (with SD 10 points) in the function subscale of the WOMAC, using Student’s t-test for independent samples, a power of 80% and an alpha value of 5%, a sample of 64 participants per study group is needed. Considering a 6-month dropout rate of 20%, about 80 individuals were needed per group (total *n* = 160).

### Statistical analysis

Descriptive statistics used mean (with SD) for quantitative variables, and absolute frequencies with percentages for qualitative variates. Baseline differences between spa therapy and control groups were examined using Fisher’s F test for independent samples or Chi-square test, as appropriate. The effects of the intervention on the primary and secondary outcomes were estimated analyzing all participants with available data. Changes in the WOMAC function subscale between baseline and 6-month (primary outcome measure) were analyzed using ANOVA for repeated measures testing the group-by-time interaction, the effect of time and the effect of the intervention (control versus spa therapy). Analyses for quantitative secondary outcomes used the same ANOVA approach. Between-group changes in qualitative outcomes were analyzed using the Chi-square test or Fisher’s exact test, as appropriate. Effect size was estimated using Cohen’s *d* and related classification: small (0.2 ≤ d < 0.5), medium (0.5 ≤ d < 0.8), and large (d ≥ 0.8). All analyses were performed using SAS version 9.4 (SAS Institute) with the significance level set at *p* ≤ 0.05.

## Results

**Fig. 1:**
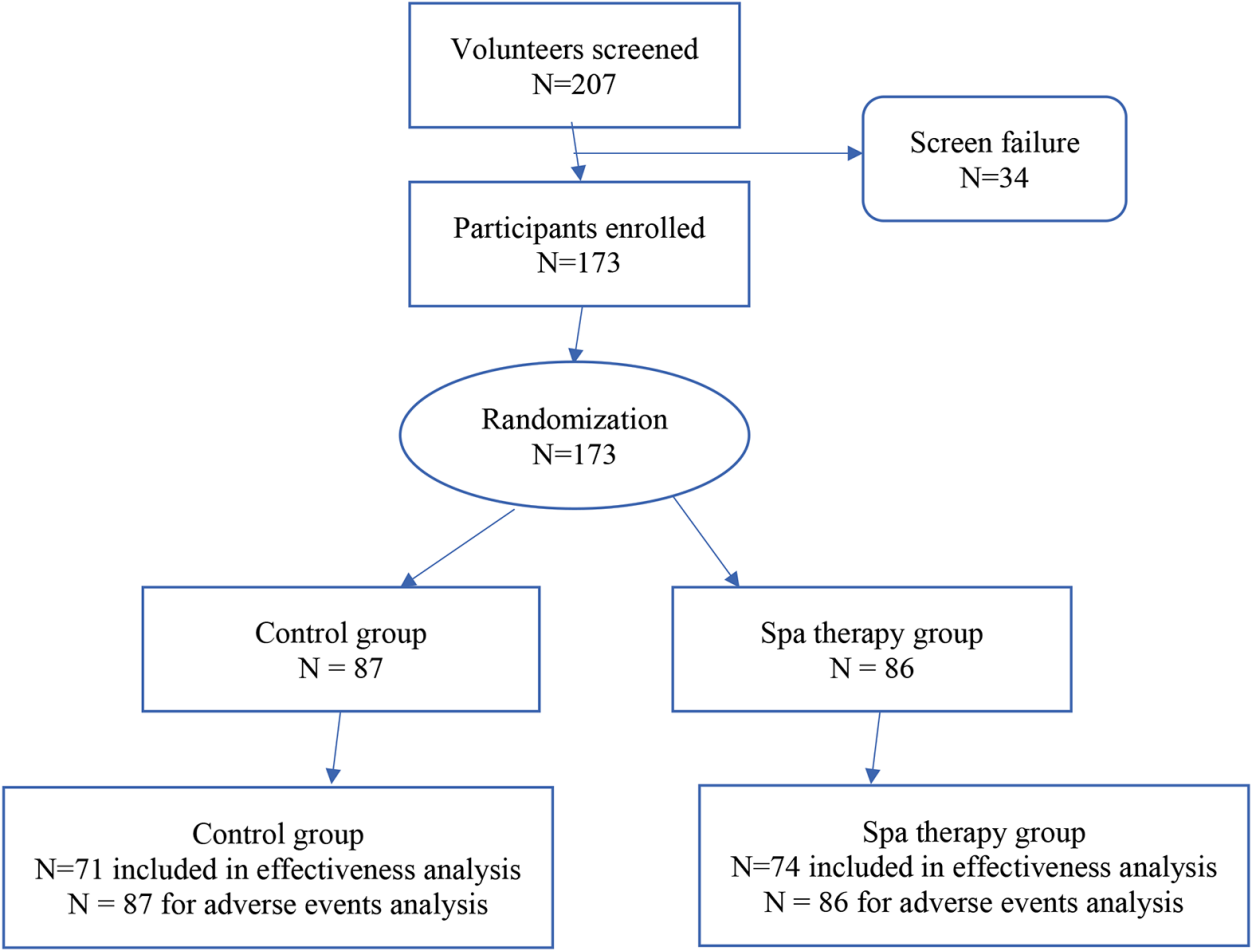
Flow chart of the study participants

This study enrolled and randomized 173 subjects (*n* = 87 in the control group; *n* = 86 in the spa therapy group). Usable data was available for 145 KOA patients, 71 controls (*n* = 69 for some secondary outcome measures due to missing data) and 74 spa therapy patients; this data was used in all the analyses presented herein. The number of and reasons for dropout did not differ between groups; main reasons for dropout were: participants no longer meeting eligibility criteria (*n* = 9; for example, improvement in the WOMAC function index between the day of enrollment and the study Day 1); withdraw of the consent (*n* = 8); lost to follow-up (*n* = 6). Figure 1 shows the flow chart of the study patients. Table 1 shows patients’ characteristics per study group. The control and spa therapy groups did not differ neither in socio-demographic and health-related (e.g., body mass index, number of diseases, blood pressure) variables, nor KOA-related variables (KOA site, Kellgren-Lawrence KOA stage, number of spa therapy before the study, percentage of people under pain control medications), except for KOA symptoms duration, which was longer for the control group.

**Table 1:** Characteristics of the study population (*n* = 145)

Variables	Control group (*n* = 71)	Spa Therapy group (*n* = 74)	*p*-value
Age, years, mean (SD)	68.3 (6.7)	67.5 (6.4)	0.49
Sex, n (%) women	46 (64.8)	50 (67.6)	0.72
Body mass index, kg/m², mean (SD)	30.1 (6.0)	29.8 (4.7)	0.78
Number of diseases (other than KOA), mean (SD)	1.9 (1.5)	1.9 (1.4)	0.94
Systolic blood pressure, mmHg, mean (SD)	139.5 (9.6)	139.7 (12.8)	0.93
Diastolic blood pressure, mmHg, mean (SD)	84.7 (8.9)	85.3 (7.9)	0.66
KOA site, n (%)			0.12
*Left*	30 (42.2)	17 (37.8)	
*Right*	21 (29.6)	28 (23)	
*Both*	20 (28.2)	29 (39.2)	
Kellgren-Lawrence KOA stage, n (%)			0.35
*Stage 2*	12 (16.9)	13 (17.6)	
*Stage 3*	32 (45.1)	25 (33.8)	
*Stage 4*	27 (38)	36 (48.6)	
Number of KOA-related spa therapy before study, mean (SD)	5.8 (5.1)	4.3 (2.8)	0.34
Pain control medications due to KOA, n (%)	58 (81.7)	60 (81.1)	0.92
KOA symptoms duration, years, mean (SD)	11.8 (7.3)	9.6 (4.9)	0.042

Compliance with spa therapy intervention was very high. Each of the 74 patients in the spa therapy group who completed the study had access to 18 days of intervention over three weeks (*n* = 1332 spa therapy sessions); only 19 sessions, involving 12 patients, were not performed, leading to an overall compliance of 98.6% (*n* = 62 patients had 100% compliance).

### Spa therapy effectiveness

#### Primary outcome measure

Table 2 shows the results of the effects of spa therapy on the WOMAC function subscale (Supplementary Material Table S1 for the 0-100 normalized score). Between-group differences showed that spa therapy improved function in average 11.7 points when compared to controls in the 6-month follow-up. The effect size according to Cohen’s *d* was 1.045, demonstrating a strong effect. Within-group comparisons found an improvement of 12.4 points in the intervention group, and a very small improvement of 0.7 points among controls. Evolution of the WOMAC function subscale over the four time-points of data collection (baseline, day 20– post-intervention, and 3- and 6-month follow-up) showed that the benefits of the intervention occurred during the three weeks of spa therapy and were sustained over six months.

**Table 2:** Effects of spa therapy on the 6-month changes in the WOMAC function (primary outcome measure) as well as changes over the whole follow-up (baseline, 20 days, 3 months, and 6 months)

Variables	Control group (*n* = 71)		Spa therapy group (*n* = 74)		Between-group mean difference (95% CI); p-value
	Mean (SD)	Within-group mean difference (95% CI); p-value^b^	Mean (SD)	Within-group mean difference (95% CI); p-value	
WOMAC function^a^					
*Baseline*	36.7 (8.7)	-	38.1 (9.2)	-	-
*6-month*	36.1 (11.8)	-0.7 (-2.9; 1.5); *p* = 0.54	25.7 (14.5)	-12.4 (-15.3; -9.4); <0.001	11.7 (8.0; 15.4); *p* < 0.0001
WOMAC function		Overall p-value = 0.76		Overall p-value < 0.001	Overall p-value < 0.001
*Baseline*	36.7 (8.7)	-	38.1 (9.2)	-	-
*Day 20 (post-intervention)*	36.8 (9.6)	0.01 (-1.5; 1.5)	25.3 (12.1)	-12.8 (-15.1; -10.5)	12.8 (10.0; 15.5)
*3-month*	36.9 (11.4)	-0.1 (-2.0; 1.8)^c^	25.7 (14)	-12.3 (-15.3; -9.4)	12.3 (8.7; 15.8)
*6-month*	36.1 (11.8)	-0.7 (-2.9; 1.5)	25.7 (14.5)	-12.4 (-15.3; -9.4)	11.7 (8.0; 15.4)

^a^This is the primary outcome measure of the study

^b^Within-group mean difference between a follow-up measure and baseline

^c^*n* = 69 in the control group for this analysis

#### Secondary outcome measures

Tables 3 and 4 display the findings on the secondary outcome measures (Supplementary Material Table S2 for WOMAC 0-100 normalized scores). The spa therapy intervention had positive effects on KOA-related pain intensity (both WOMAC pain subscale and 0-100 pain visual analog scale), stiffness, and overall symptoms severity, as well as on patients’ quality of life (Table 3). Similar to the findings on the primary outcome measure, these benefits occurred during the 3-week period of spa therapy intervention and were sustained through the 3-month and 6-month length of follow-up. Indeed, patients in the control group showed stability over time on the scores of the WOMAC pain and stiffness subscales and on the WOMAC total score (no significant within-group changes), while those in the spa therapy group significantly decreased their scores. This resulted in a between-group mean difference of about 1.7, 4.8 and 19.3 points for WOMAC stiffness, pain and total scores, respectively, favoring the spa group between baseline and post-intervention (time-point of data collection “day 20”). Similar findings were found for quality of life (no changes in controls and improvement on spa therapy), leading to a between-group mean difference favoring the intervention group. For the pain visual analog scale, whereas controls increased, the spa group decreased pain levels significantly, resulting in a between-group mean difference of about 23.4 mm favoring the spa group (between baseline and post-intervention).

**Table 3:** Effects of spa therapy on the changes in the KOA symptoms and severity and patients’ quality of life over the whole follow-up (baseline, 20 days, 3 months, and 6 months)

Variables	Control group (*n* = 71)		Spa therapy group (*n* = 74)		Between-group mean difference (95% CI); p-value
	Mean (SD)	Within-group mean difference (95% CI); p-value	Mean (SD)	Within-group mean difference (95% CI); p-value	
WOMAC total score (0–96)		Overall p-value = 0.82		Overall p-value < 0.001	Overall p-value < 0.001
*Baseline*	52.1 (11.3)	-	54.7 (12.4)	-	-
*Day 20 (post-intervention)*	52.1 (12.6)	0.2 (-1.6; 2.1)	35.6 (16.4)	-19.1 (-22.2; -16.0)	19.3 (15.7; 22.9)
*3-month*	52.0 (15.5)	-0.1 (-2.7; 2.5)^a^	36.6 (19.2)	-18.1 (-22.1; -14.2)	18.0 (13.2; 22.8)
*6-month*	51.1 (15.8)	-0.8 (-3.7; 2.2)	36.9 (19.7)	-17.8 (-21.9; -13.8)	17.1 (12.1; 22.1)
WOMAC pain (0–20)		Overall p-value = 0.72		Overall p-value < 0.001	Overall p-value < 0.001
*Baseline*	10.4 (2.4)	-	11.5 (2.9)	-	-
*Day 20 (post-intervention)*	10.7 (2.5)	0.3 (-0.1; 0.8)	7.0 (3.3)	-4.5 (-5.1; -3.8)	4.8 (4.0; 5.6)
*3-month*	10.6 (3.3)	0.2 (-0.5; 0.8)	7.4 (4.1)	-4.1 (-5.0; -3.2)	4.2 (3.1; 5.3)
*6-month*	10.4 (3.1)	-0.1 (-0.7; 0.6)	7.6 (4.1)	-3.9 (-4.8; -2.9)	3.8 (2.6; 4.9)
WOMAC stiffness (0–8)		Overall p-value = 0.84		Overall p-value < 0.001	Overall p-value < 0.001
*Baseline*	4.6 (1.3)	-	5.1 (1.4)	-	-
*Day 20 (post-intervention)*	4.5 (1.4)	-0.1 (-0.3; 0.1)	3.3 (1.6)	-1.8 (-2.2; -1.5)	1.7 (1.3; 2.2)
*3-month*	4.5 (1.7)	-0.1 (-0.5; 0.2)^b^	3.4 (1.7)	-1.7 (-2.2; -1.3)	1.6 (1.1; 2.2)
*6-month*	4.5 (1.3)	-0.04 (-0.4; 0.3)	3.5 (1.8)	-1.6 (-2.0; -1.2)	1.6 (1.0; 2.1)
Pain visual analog scale (0-100)		Overall p-value < 0.001		Overall p-value < 0.001	Overall p-value < 0.001
*Baseline*	47.0 (18.8)	-	55.2 (17.7)	-	-
*Day 20 (post-intervention)*	51.1 (16.6)	3.9 (-0.1; 7.9)	35.7 (20.8)	-19.5 (-24.2; -14.9)	23.4 (17.3; 29.5)
*3-month*	55.6 (16.7)	8.6 (4.0; 13.3)^b^	42.8 (24.8)	-12.5 (-18.4; -6.5)	21.1 (13.6; 28.6)
*6-month*	55.7 (19)	8.5 (3.3; 13.7)	46.8 (23.1)	-8.8 (-14.7; -3.0)^c^	17.4 (9.6; 25.1)
EQ-5D-3 L		Overall p-value = 0.24		Overall p-value < 0.001	Overall p-value < 0.001
*Baseline*	0.58 (0.23)	-	0.55 (0.23)	-	-
*Day 20 (post-intervention)*	0.59 (0.20)	0.01 (-0.03; 0.06)	0.75 (0.17)	0.2 (0.15; 0.25)	-0.19 (-0.26; -0.12)
*3-month*	0.54 (0.26)	-0.04 (0.1; 0.02)^b^	0.70 (0.23)	0.15 (0.1; 0.2)	-0.19 (-0.27; -0.11)
*6-month*	0.58 (0.25)	-0.001 (-0.04: 0.04)	0.71 (0.23)	0.16 (0.10; 0.21)	-0.16 (-0.23; -0.09)

^a^*n*=69 in the control group for this analysis

^b^*n* = 70 in the control group for this analysis

^c^*n* = 73 in the intervention group for this analysis

**Table 4:** Effects of spa therapy on the changes in clinically meaningful changes in KOA symptoms, patients’ perception of KOA symptoms’ evolution, KOA treatments, pain control medications, and KOA-related care services utilization (lower-body surgery, hospitalizations of > 24 h, and consultations with a physician - general practitioner or specialist) over the whole follow-up (20 days, 3 months, and 6 months)

Variables	Day 20 (post-intervention)			3 months			6 months		
	Control group (*n* = 69)	Spa Therapy (*n* = 74)	p-value	Control group (*n* = 69)	Spa Therapy (*n* = 74)	p-value	Control group (*n* = 69)	Spa Therapy (*n* = 74)	p-value
Clinically meaningful changes in KOA symptoms, n (%)	13 (18.8%)	58 (78.4%)	< 0.001	14 (20.3%)	51 (68.9%)	< 0.001	14 (20.3%)	54 (73%)	< 0.001
Patients’ perception of KOA symptoms’ evolution compared to baseline, n (%)			< 0.001			< 0.001			< 0.001
*Highly improved*	0 (0%)	12 (16.2%)		1 (1.4%)	13 (17.6%)		0 (0%)	7 (9.5%)	
*Moderately improved*	2 (2.9%)	34 (45.9%)		0 (0%)	20 (27%)		3 (4.3%)	24 (32.4%)	
*Mildly improved*	3 (4.3%)	24 (32.4%)		4 (5.7%)	29 (39.2%)		3 (4.3%)	30 (40.5%)	
*Did not change*	52 (74.3%)	3 (4.1%)		48 (68.6%)	10 (13.5%)		43 (61.4%)	8 (10.8%)	
*Mildly worsened*	12 (17.1%)	1 (1.4%)		15 (21.4%)	2 (2.7%)		17 (24.3%)	3 (4.1%)	
*Moderately worsened*	0 (0%)	0 (0%)		2 (2.9%)	0 (0%)		3 (4.3%)	2 (2.7%)	
*Highly worsened*	1 (1.4%)	0 (0%)		0 (0%)	0 (0%)		1 (1.4%)	0 (0%)	
Concomitant treatments for KOA, n (%)^a^	4 (5.6%)	0 (0%)	0.055	6 (8.5%)	2 (2.7%)	0.16	2 (2.8%)	4 (5.4%)	0.68
KOA pain control medication, n (%)^b^	24 (33.8%)	9 (12.2%)	0.003	33 (46.5%)	17 (23%)	0.003	32 (45.1%)	20 (27%)	0.03
Consultations with a physician, n (%)^c^	6 (8.6%)	2 (2.7%)	0.16	16 (22.9%)	8 (10.8%)	0.07	20 (28.6%)	13 (17.6%)	0.16

^a^At baseline, 12 (16.9%) and 14 (18.9%) individuals in the control and spa therapy groups (*p* = 0.83), respectively, had at least one KOA treatment (pharmacological or non-pharmacological) in addition to usual pain control medications

^b^At baseline, 58 (81.7%) and 60 (81.1%) individuals in the control and spa therapy groups (*p* = 1), respectively, had at least one pain control medication for KOA treatment

^c^At baseline, 8 (11.4%) and 6 (8.1%) individuals in the control and spa therapy groups (*p* = 0.58), respectively, had at least one KOA-related consultation with a physician (general practitioner or specialist)

Furthermore, the number of patients with a clinically meaningful change in KOA symptoms was higher in the spa therapy group compared to controls at all follow-up time-points (Table 4). Similarly, compared to controls, the proportion of participants under pain control drugs was lower whereas the proportion of people perceiving that their KOA symptoms improved was higher in the spa group. No differences between groups were found for the proportion of individuals receiving KOA-related concomitant treatments or for the proportion of those having consultations with a physician during the follow-up.

### Adverse events

In total, 59 adverse events occurred during the 6-month length of the study, being 33 among controls and 26 in the spa therapy group. Only four serious events were identified: 3 in the spa therapy (2 knee prothesis; 1 prostate cancer), and 1 in controls (1 knee prothesis). Among the 26 events in the spa therapy group, four occurred during spa treatment: 3 of them were considered as possibly associated to the intervention (moderate orthostatic arterial hypotension after the bath; mild itching; and moderate painful flare-up related to the patient’s stress during spa care), and one as probably related to the intervention (mild pruritus); all of them were resolved without any specific treatment, without sequelae.

## Discussion

This RCT showed that, when compared to usual care, three weeks of spa therapy intervention (mainly composed of baths, underwater massage, and mud poultice) was effective in improving mobility and functional ability (as measured by the WOMAC function subscale over a 6-month follow-up) in adults 50-to-80 years-old with KOA. Moreover, compared to controls, spa therapy produced positive effects on several important KOA-related clinical outcomes, including overall severity of symptoms (WOMAC total scores), pain (both WOMAC pain subscale and pain visual analog scale), stiffness (WOMAC stiffness subscale), and on patient’s quality of life. Participants in the spa therapy group more often had both clinically meaningful improvements in KOA-symptoms and the self-perception their KOA-symptoms were improved, and less often used pain-management drugs than in the control group. All these benefits occurred without an increase in adverse events.

The improvements in function (WOMAC subscale) in our study are equivalent or higher than those of previous RCT testing the effects of spa therapy in patients with KOA (Forestier et al. 2010; Özkuk et al. 2017, 2018). Similarly, the magnitude of improvements in pain and stiffness (WOMAC subscales and pain visual analog scale) as well as in overall severity of KOA symptoms (WOMAC total scores) are comparable to those found in other trials (Fioravanti et al. 2015; Fraioli et al. 2018; Özkuk et al. 2017; Varzaityte et al. 2020; Yaşar et al. 2021). The improvements in KOA-related symptoms in our study may be considered clinically meaningful. Indeed, there is evidence that improvements of 12.5% and 16% (Conaghan et al. 2022) are clinically relevant for all the WOMAC subscales as well as the WOMAC total score: in our study, all WOMAC outcomes improved by about 30% in the spa therapy group between baseline and post-intervention (at the end of the 3-week intervention period). Moreover, changes in the pain visual analog scale after the spa therapy intervention were also clinically relevant in our study since the between-group mean difference favoring spa therapy at the end of the 3-week intervention was higher than 19.9 mm, the minimum clinically meaningful change for patients with KOA (Tubach et al. 2005). Regarding pain assessments, the reasons why controls worsened on pain visual analog scale but not on WOMAC pain subscale remain unknown and deserves further investigation, although it is reasonable to think that the visual analog scale is more sensitive to change. More than 70% of individuals in the spa therapy group had a clinically meaningful improvement after the 3-week intervention as measured by a pain reduction ≥ 19.9 mm in the visual analog scale or a decrease > 9.1 in the WOMAC function subscale (0-100 normalized score), whereas they were only about 20% in the control group. In addition, we found that patient reported outcomes about KOA-related symptoms favored the intervention group, reinforcing the findings that the spa therapy resulted in clinically relevant improvements. Interestingly, our findings clearly demonstrated that most of the spa therapy benefits occurred during the 3-week intervention and remained over 6 months, results that can have important clinical implications. Indeed, they confirm that a relatively short-time intervention may have medium-to-long-term clinical benefits, suggesting that no more than two intervention periods per year, 6-month apart, would be sufficient to treat KOA symptoms in most patients at Kellgren-Lawrence stages 2–4. A longer trial would contribute to establish the effectiveness of spa therapy intervention over the whole year or still longer; there is scarce evidence pointing out that some of the spa therapy benefits may remain after a 9-month period of follow-up (Forestier et al. 2010).

Taken together, our findings demonstrate that spa therapy, on one hand, benefits function, pain and quality of life in a clinically meaningful way, and on the other hand, decreases pain-control drug use. Our positive results on quality of life corroborate the literature on this topic (Antonelli et al. 2018). They also suggest the spa therapy might be cost-effective. Indeed, quality of life scales are often used as an economic measure through the assessment of quality-adjusted life years, particularly in the field of osteoarthritis (Østerås et al. 2024; Reinhold et al. 2008). Therefore, spa therapy has the potential to reduce the burden of KOA to the patients themselves and their family, but also to the society by reducing KOA-associated costs. Future investigations in this field should examine if the clinical effectiveness of spa therapy also leads to KOA-related costs savings.

The main strengths of this study are the RCT design, the well-powered and relatively large sample, and an important compliance rate regarding the spa therapy, which allowed us to demonstrate the clinical efficacy of the intervention. The main limitations involve the absence of cost-effectiveness investigations, and the absence of a longer follow-up. The former limitation precluded us to affirm the intervention is cost-saving and that it reduces the KOA-related economic burden to patients and society; whereas the latter impeded us to examine for how long the patients keep clinically meaningful benefits from the spa therapy intervention. Another limitation, which is inherent to nearly all non-pharmacological interventions (Boutron et al. 2004), is the quasi-impossibility of blinding participants to group allocation. The extent to which this may have impacted the results remains unknown. Furthermore, the control group was a waiting list group, which may have a negative impact on outcome measures by leading to an overestimation of the intervention effects (Cunningham et al. 2013). The dropout rate observed in our study (16.2%) was similar to that of other spa therapy RCT in people with KOA (Fioravanti et al. 2015; Forestier et al. 2010).

## Conclusions

This RCT demonstrated the effectiveness of a 3-week spa therapy to reduce the symptoms of KOA and to improve quality of life in patients aged 50–80 years with KOA at Kellgren-Lawrence stages 2–4. The positive effects were evident from the end of the 3-week intervention period and were sustained through the 6-month follow-up. A longer RCT, investigating cost-effectiveness and other healthcare cost analysis, is needed to contribute to establishing spa therapy as a treatment of choice for KOA and to define the most appropriate frequency for this intervention. Investigating the mechanisms through which spa therapy positively affects KOA symptoms is also important and could inform for the development of future treatments in the field.

## Electronic supplementary material

Below is the link to the electronic supplementary material.

Supplementary Material 1

## Data Availability

https://www.springernature.com/gp/authors/research-data-policy/data-availability-statements/12330880). Access to data can be provided upon reasonable request to the corresponding author and validation of the study scientific committee.
