# Supplementary material for: The effectiveness of a 3-week spa therapy on the 6-month mobility and functional ability of patients with knee osteoarthritis: the ANGELLO randomized controlled trial
Source: Int J Biometeorol. 2025 May 13;69(7):1715–24. doi: 10.1007/s00484-025-02927-2 (PMC12178974; doi:10.1007/s00484-025-02927-2)
Supplement: Supplementary file 1 — Supplementary Material 1 [file 484_2025_2927_MOESM1_ESM.docx]

Supplementary Materials

Table S1. Effects of Spa therapy on the 6-month changes in the WOMAC function normalized score (0 – 100, higher is worse) as well as changes over the whole follow-up (baseline, 20 days, 3 months, and 6 months).

| Variables | Control group (n=71) | | Spa therapy group (n=74) | |  |
| --- | --- | --- | --- | --- | --- |
|  | Mean (SD) | Within-group mean difference (95% CI); p-value^a^ | Mean (SD) | Within-group mean difference (95% CI); p-value | Between-group mean difference (95% CI); p-value |
| WOMAC function normalized |  |  |  |  |  |
| *Baseline* | 54.0 (12.7) | - | 56.0 (13.6) | - | - |
| *6-month* | 53.0 (17.4) | -1.0 (-4.2 ; 2.2); p=0.54 | 37.8 (21.3) | -18.2 (-22.5 ; -13.8); <0.001 | 17.2 (11.8 ; 22.6) ; p < 0.0001 |
|  | | | | | |
| WOMAC function normalized |  | Overall p-value = 0.76 |  | Overall p-value < 0.001 | Overall p-value < 0.001 |
| *Baseline* | 54.5 (12.6) | - | 56.0 (13.6) | - | - |
| *Day 20 (post-intervention)* | 54.2 (14.1) | 0.02 (-2.2 ; 2.2) | 37.2 (17.8) | -18.8 (-22.1 ; -15.4) | 18.8 (14.8 ; 22.8) |
| *3-month* | 54.3 (16.7) | -0.1 (-3.0 ; 2.7)^b^ | 37.8 (20.6) | -18.1 (-22.4 ; -13.8) | 18.0 (12.8 ; 22.2) |
| *6-month* | 53.1 (17.6) | -1.0 (-4.2 ; 2.2) | 37.8 (21.3) | -18.2 (-22.5 ; -13.8) | 17.2 (11.8 ; 22.6) |

^a^Within-group mean difference between a follow-up measure and baseline

^b^ n=69 in the control group for this analysis

Table S2. Effects of spa therapy on the changes in the KOA symptoms and severity (normalized 0-100 scores, higher is worse) during the whole follow-up (baseline, 20 days, 3 months, and 6 months).

| Variables | Control group (n=71) | | Spa therapy group (n=74) | |  |
| --- | --- | --- | --- | --- | --- |
|  | Mean (SD) | Within-group mean difference (95% CI); p-value | Mean (SD) | Within-group mean difference (95% CI); p-value | Between-group mean difference (95% CI); p-value |
| WOMAC total score normalized |  | Overall p-value = 0.82 |  | Overall p-value < 0.001 | Overall p-value < 0.001 |
| *Baseline* | 54.3 (11.7) | - | 57.0 (12.9) | - | - |
| *Day 20 (post-intervention)* | 54.2 (13.2) | 0.2 (-1.7 ; 2.2) | 37.1 (17.1) | -19.8 (-23.1 ; -16.6) | 20.1 (16.3 ; 23.9) |
| *3-month* | 54.1 (16.2) | -0.2 (-2.9 ; 2.6)^a^ | 38.1 (20.0) | -18.9 (-23.0 ; -14.8) | 18.8 (13.8 ; 23.7) |
| *6-month* | 53.2 (16.4) | -0.8 (-3.9 ; 2.3) | 38.4 (20.5) | -18.6 (-22.8 ; -14.4) | 17.8 (12.6 ; 23.0) |
| WOMAC pain normalized |  | Overall p-value = 0.72 |  | Overall p-value < 0.001 | Overall p-value < 0.001 |
| *Baseline* | 52.2 (11.9) | - | 57.4 (14.3) | - | - |
| *Day 20 (post-intervention)* | 53.6 (12.6) | 1.7 (-0.6 ; 4.0) | 35.1 (16.4) | -22.3 (-25.7 ; -18.9) | 24.0 (19.9 ; 28.1) |
| *3-month* | 53.0 (16.3) | 0.8 (-2.4 ; 3.9) | 37.1 (20.4) | -20.3 (-24.9 ; -15.8) | 21.1 (15.6 ; 26.7) |
| *6-month* | 52.1 (15.6) | -0.3 (-3.6 ; 3.1) | 38.2 (20.5) | -19.3 (-23.9 ; -14.6) | 19.0 (13.2 ; 24.7) |
| WOMAC stiffness normalized |  | Overall p-value = 0.84 |  | Overall p-value < 0.001 | Overall p-value < 0.001 |
| *Baseline* | 57.3 (16.7) | - | 64.4 (16.9) | - | - |
| *Day 20 (post-intervention)* | 55.7 (17.1) | -1.4 (-4.2 ; 1.4) | 41.4 (19.8) | -23.0 (-27.6 ; -18.4) | 21.6 (16.2 ; 26.9) |
| *3-month* | 55.7 (20.7) | -1.6 (-6.2 ; 3.0)^b^ | 42.6 (21.4) | -21.8 (-26.9 ; -16.7) | 20.2 (13.4 ; 27.0) |
| *6-month* | 56.3 (16.5) | -0.5 (-5.0 ; 3.9) | 44.1 (22.3) | -20.3 (-25.3 ; -15.2) | 19.7 (13.0 ; 26.4) |

^a^ n=69 in the control group for this analysis

^b^ n=70 in the control group for this analysis
